# Complex Variation in Measures of General Intelligence and Cognitive Change

**DOI:** 10.1371/journal.pone.0081189

**Published:** 2013-12-12

**Authors:** Suzanne J. Rowe, Amy Rowlatt, Gail Davies, Sarah E. Harris, David J. Porteous, David C. Liewald, Geraldine McNeill, John M. Starr, Ian J. Deary, Albert Tenesa

**Affiliations:** 1 The Roslin Institute, The University of Edinburgh, Roslin, Scotland, United Kingdom; 2 Centre for Cognitive Ageing and Cognitive Epidemiology, University of Edinburgh, Edinburgh, Scotland, United Kingdom; 3 Medical Genetics Section, The University of Edinburgh, Edinburgh, Scotland, United Kingdom; 4 Institute of Applied Health Sciences, University of Aberdeen, Aberdeen, Scotland, United Kingdom; 5 Alzheimer Scotland Dementia Research Centre, The University of Edinburgh, Edinburgh, Scotland, United Kingdom; 6 Department of Psychology, University of Edinburgh, Edinburgh, Scotland, United Kingdom; 7 Medical Research Council Human Genetics Unit at the Medical Research Council Institute of Genetics and Molecular Medicine, University of Edinburgh, Edinburgh, Scotland, United Kingdom; Yale University, United States of America

## Abstract

Combining information from multiple SNPs may capture a greater amount of genetic variation than from the sum of individual SNP effects and help identifying missing heritability. Regions may capture variation from multiple common variants of small effect, multiple rare variants or a combination of both. We describe regional heritability mapping of human cognition. Measures of crystallised (g_c_) and fluid intelligence (g_f_) in late adulthood (64–79 years) were available for 1806 individuals genotyped for 549,692 autosomal single nucleotide polymorphisms (SNPs). The same individuals were tested at age 11, enabling us the rare opportunity to measure cognitive change across most of their lifespan. 547,750 SNPs ranked by position are divided into 10, 908 overlapping regions of 101 SNPs to estimate the genetic variance each region explains, an approach that resembles classical linkage methods. We also estimate the genetic variation explained by individual autosomes and by SNPs within genes. Empirical significance thresholds are estimated separately for each trait from whole genome scans of 500 permutated data sets. The 5% significance threshold for the likelihood ratio test of a single region ranged from 17–17.5 for the three traits. This is the equivalent to nominal significance under the expectation of a chi-squared distribution (between 1df and 0) of P<1.44×10^−5^. These thresholds indicate that the distribution of the likelihood ratio test from this type of variance component analysis should be estimated empirically. Furthermore, we show that estimates of variation explained by these regions can be grossly overestimated. After applying permutation thresholds, a region for g_f_ on chromosome 5 spanning the *PRRC1* gene is significant at a genome-wide 10% empirical threshold. Analysis of gene methylation on the temporal cortex provides support for the association of PRRC1 and fluid intelligence (P = 0.004), and provides a prime candidate gene for high throughput sequencing of these uniquely informative cohorts.

## Introduction

Loss of cognitive function is one of the most feared aspects of growing old. Intelligence and the rate of age related cognitive change vary widely in healthy individuals and have been associated with health status, longevity and quality of life [1], [2], [3], [4], [5], [6]. As the general population ages, cognitive health is of paramount importance, and understanding the underlying mechanisms of general intelligence and age-related decline has wide-ranging social and economic implications. Although pathological cognitive decline has been studied in diseases such as Alzheimer's [7], available phenotypic measures for lifetime changes in cognitive abilities of healthy individuals are rare. An important part of the variation in human general intelligence and in non-pathological, age-associated cognitive decline [8], [9] can be attributed to heritable genetic variation. Identifying the genes and loci that contribute to the estimated genetic variance would offer new biological insight, with opportunities to develop tailored interventions and to inform policy makers.

Here we analyse the genetic contributions to complex variation in three measures of intelligence: (i) crystallised intelligence; (ii) fluid general intelligence; and (iii) lifetime change in intelligence. We use three Scottish birth cohorts whose intelligence was measured in childhood (age 11 years) and again in late adulthood (age 65 to 79 years). Crystallised intelligence (*g*_c_) is typically assessed using vocabulary and knowledge-based tests, and tends to remain stable with age. Fluid intelligence (*g*_f_) is assessed using tests that require on-the-spot thinking — often with abstract materials and under time pressure — and tends to peak in early adulthood and decline thereafter [10], [11]. Here, cognitive change was measured as fluid intelligence in old age adjusted for intelligence measured at age 11 as described in Deary et al. [9], who showed, using the same data, that the lower bound estimate for the proportion of variation in lifetime change of intelligence explained by genetic factors was 0.24.

To date, as is seen in many complex traits, and despite moderate-to-high heritability estimates, genomic studies have yielded little knowledge of the underlying genetic factors affecting cognitive traits. Although studies for other complex traits have been successful at garnering the few common genetic variants that explain a sizeable amount of variation, genome wide association studies (GWAS) have generally failed to capture a large proportion of the genetic variation in complex traits [12], [13], [14]. A recent GWAS for crystallised and fluid intelligence did not result in any replicable genome-wide significant association despite moderately high heritability estimates of 0.4 (s.e. 0.11) and 0.51(s.e. 0.11) for *g*_c_ and *g*_f_ respectively for the population under study [8]. To address this gap, we have applied a recently proposed analytical approach [15] that captures the combined effect of multiple genetic variants at a region of the genome, thereby identifying some of the heritability missing when applying standard ‘one at a time’ SNP analyses [16], [17]. This approach has the potential to overcome stringent multiple testing penalties and has been shown to be more powerful than the ‘one at a time’ SNP approach in simulated and real data [15]. We hypothesise that combinations of common and rare variants, that are not in complete LD with common tagging SNPs, may account for a substantial part of the missing heritability and that these will be best captured by estimating the genetic variation from an entire ‘region’ or geographically co-located set of SNPs. The trade-off comes between capturing as much variation as possible, whilst having the resolution to locate causal effects. Here we divide the genome in two ways (regionally and functionally): firstly, into overlapping regions of 101 SNPs; and secondly by chromosome, separating SNPs that lie within genes and SNPs that map outside a 5 kb boundary of genes. We examine the genetic variation explained by each region or chromosome for crystallised and fluid intelligence and for the lifetime change in fluid intelligence, and we compare that to the most significant results obtained from the ‘one SNP at a time’ association approach.

## Materials and Methods

### Phenotypic Data

Ethical approval for all the projects was obtained from the Lothian Research Ethics Committee. Data were gathered from three longitudinal studies of relatively healthy older individuals with detailed cognitive phenotypes: the Lothian Birth Cohorts of 1921 (LBC1921, N = 550) and 1936 (LBC1936, N = 1091), and the Aberdeen Birth Cohort of 1936 (ABC1936, N = 548). The years 1921 and 1936 refer to the participant's year of birth. Participants took a validated intelligence test at a mean age of 11 years: the Moray House Test No. 12 (MHT), which is a test of general intelligence [18], [19] and detailed follow-up assessments at a mean age (sd) of 79.1 (0.6), 69.5 (0.8) and 64.6 (0.9) for LBC1921, LBC1936 and ABC1936, respectively. Cognitive test scores from age 11 and old age were available.

### Construction of phenotypes

Selection of individuals, ethical consent, and full details of the assessments have been described in previous studies [8], [9], [18], [19], [20], [21]. In brief, for each cohort, cognitive phenotypes of fluid-type and crystallized-type intelligence were constructed [19], [20]. The final measure of lifetime cognitive change was constructed by adjusting fluid intelligence in old age for prior cognitive ability providing a quantitative measure of cognitive change from age 11 to old age. Phenotypes were adjusted within cohort for age and standardised within gender, and are further defined in Appendix 1.

### Genotypic data

Following informed consent, venesected whole blood was collected for DNA extraction. A total of 599,011 single nucleotide polymorphisms (SNPs) were genotyped using the Illumina610-Quadv1 chip as described previously [8]. Quality control (QC) procedures were performed per SNP and per sample. Individuals were excluded from further analysis if genetic and reported gender did not agree. Samples with a call rate ≤0.95, and those showing evidence of non-European descent by multidimensional scaling analysis, were also removed. SNPs were included in the analyses if they met the following conditions: call rate ≥0.98, minor allele frequency ≥0.01, and Hardy-Weinberg equilibrium test with p≥0.001. To avoid bias from hidden family structure, if a pair of individuals shared more than 2.5% of the genome in common, one individual was omitted from the analysis. After QC, 1804 individuals (ABC1936, N = 376; LBC1921, N = 484; LBC1936, N = 944), and 547,750 autosomal SNPs were included in the analysis.

### Estimation of regional and functional genetic contribution

In a population of unrelated individuals, SNP genotypes can be used to estimate shared co-ancestry or identity by state between individuals with rare SNPs weighted more heavily. Under certain assumptions it can be shown that a region that is shown to be identical by state will also be identical by descent [22]. The *n×n* genomic relationship matrix (GRM) of relatedness at a population level between *n* individuals gives the covariance structure for the phenotype based on the premise that the more related two individuals are, or the greater the amount of the genome they share in common, the greater the expectation of phenotypic similarity.

Using theory adapted from standard variance components or pedigree based linkage analysis [23], [24], [25] and further developed for genomic prediction [26], [27], [28], a GRM containing information from the genotypes of *m* SNPs can be used to solve a linear mixed model [Model 1] and partition the phenotypic variance into estimates of the genetic and environmental variance [15], [29]. To avoid confusion with the well-known family-based estimates of heritability [30] we define the amount of phenotypic variance captured by the genotypes of unrelated individuals as population-sense heritability (h^2^_ps_). The linear mixed model (LMM) is:
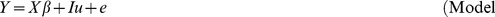
(Model \ 1)Where **Y** is an *n×1* vector of phenotypes for *n* individuals; **X** ***_n×21_*** is the incidence matrix relating the regression coefficients for 20 principal components and gender to the *n* individuals; **β** is a *21×1* vector of fixed effects; **u** is a *n×1* vector of the additive genomic random effects where *u∼N(0,Gσ^2^_u_)*, **G** is an *n×n* genomic relationship matrix estimated from the SNP genotypes and σ**^2^_u_** is the genetic variance captured by the SNPs used to estimate the relationships among the *n* individuals; **I** is an *n×n* identity matrix; and **e** is an *n×1* vector of individual residual effects. The variance of **Y** is *var (Y) = Gσ* ***^2^*** *_u_+Iσ* ***^2^*** *_e_*. **G** is calculated following Van Raden (2008) [28]. In short, an *n×m* matrix, **W**, is constructed where *m* is the number of SNPs available. The elements of **W**, *w_ij_*, are defined as 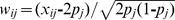 with *x_ij_* being 0, 1 or 2 for the three possible SNP genotypes for the *j*-th SNP of the *i*-th individual and 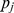 being the allele frequency of the *j*-th SNP. **G** is calculated as *WW'/m*.

An extension of this to a bivariate analysis [Model 2] was used to estimate phenotypic and genetic covariances amongst measures of intelligence.
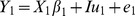


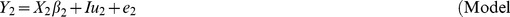
(Model\ 2)Where 1 and 2 refer to trait 1 and trait 2, **u**_1_ and **u**_2_ are *n×1* vectors of additive genomic random effects. **G** is the genomic relationship matrix between all individuals as described above. The additive genetic covariance of **Y_1_** and **Y_2_** - cov(**u**_1_, **u** *_2_*) = σ^2^_u12_ and the environmental covariance cov (**e_1_**, **e_2_**) is σ^2^_e12_. The additive genetic correlation of Y_1_ and Y_2_ is σ^2^_u12_/σ_u1_ σ_u2_, and the variance-covariance matrix for **Y = [Y_1_**, **Y_2_]** is 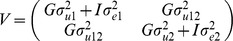. A full derivation of the estimation of the genetic covariance is given in [31].

### Regional population-sense heritability

Yang et al. [32] implement the linear mixed model [Model 1] in the software package GCTA and have shown that the method can be used to partition the genetic variation across chromosomes and functional regions of the genome such as genes [15].

By combining information on multiple SNPs within a genomic region we aim to capture a substantial part of the heritability missed by traditional ‘one SNP at a time’ approaches. Identifying those regions of the genome that capture most variation is an efficient way of selecting candidate regions for high throughput sequencing that could complement whole-exome sequencing experiments until whole genome sequencing is feasible for large numbers of samples. Here, autosomal SNPs were ranked by genomic location and divided into regions spanning 101 consecutive SNPs. Regions were overlapping to allow for the possibility that genetic variation is distributed among two or more windows, with a shared region between two consecutive regions spanning 50 SNPs, resulting in 10,908 overlapping regions from 547,750 SNPs. Each region was fitted individually in the linear mixed model [Model 3].
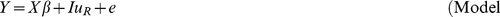
(Model\ 3)Where R is the genomic region. **u** *_R_* is a vector of *n* additive genomic random effects from the region, *n* is the number of individuals and **I** is the identity matrix as described above. 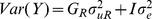; where **G_R_** is a GRM derived only from SNPs within the defined region.

### Functional population-sense heritability

Genes are the most important functional units of the genome. In order to investigate their contribution to variation in cognition we partitioned, for each of the autosomes, the genetic variance captured by SNPs located inside and outside genes. SNPs mapping to each autosome were separated into those that mapped within 5 kb of the transcription start and end sites of a gene (i.e. within genes) and those that mapped outside these limits. Genome build 37 was used to identify genes and gene limits. A linear mixed model was used to fit forty-four variance components simultaneously, capturing SNPs within genes and SNPs outside genes on each of the 22 human autosomes [Model 4].
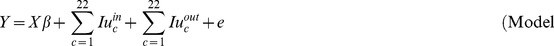
(Model\ 4)Where **u** ***_c_^in^*** is the vector of additive genomic random effects which for each chromosome is solved using a GRM derived from SNPs which lie within genes or within a 5 kb boundary of a gene on that chromosome *c*; **u** ***_c_^out^*** is a vector of additive genomic random effects solved using a GRM derived from SNPs which lie outside genes on that chromosome *c*.

For comparison we grouped SNPs by chromosome and the population-sense heritability was estimated for individual chromosomes [Model 5]. This approach was used previously in a meta-analysis of five cohorts including those described here for adult fluid and crystallised intelligence [8] but not for cognitive change.
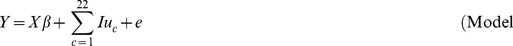
(Model\ 5)Where **u** *_c_* is the vector of additive genomic random effects on chromosome *c* solved for each chromosome using a GRM derived from SNPs which lie on that chromosome *c*.

### Model fitting

Initially all SNPs were fitted in the model to estimate the genetic variance and overall heritability for the three cognitive traits in the population. Bivariate analyses to estimate covariances amongst the three cognitive measures were performed using ASReml 2 software [33]. To avoid confounding of genetic variation of the trait and potential variation due to population stratification, eigenvectors were estimated from the genetic relationship matrix and the first 20 principal components were fitted as covariates in the linear mixed model. Sex was also fitted into the model. Analyses were subsequently carried out fitting the regions defined above to estimate regional and functional population-sense heritability.

GCTA/ACTA [34] solves the LMM and obtains estimates of genetic and residual variances by restricted maximum likelihood (REML) using the average information (AI) algorithm.

Test statistics were obtained using a standard likelihood ratio test (LRT) statistic calculated as twice the difference between the log likelihoods of the full model and a null or reduced model that did not fit a genetic component. For a single test, the expectation of the LRT for testing one extra variance component is a 50∶50 mixture of a point mass of 0 and a chi square distribution with 1df [35]. This is so because under the null hypothesis the true value of the variance components is on the boundary of the parameter space defined by the alternative hypothesis.

Results from the 10,908 regions were ranked by likelihood ratio test statistic. The top ten non –overlapping or approximately 0.1% of regions were fitted back into a linear model with an eleventh ‘polygenic’ variance component comprising all the available autosomal SNPs. This model was tested against a null model containing only the polygenic variance component under the expectation that the likelihood ratio test is distributed as a chi-square with ten degrees of freedom. We repeated the analyses without the ‘polygenic’ variance component and obtained virtually the same results.

Finally, the contribution of the identified top ten regions for each of the traits were analysed for putative pleiotropic effects across cognitive phenotypes.

### Permutation analysis

To date there is little evidence for the empirical distribution of a suitable threshold for the LRT statistic when testing multiple genomic regions. Rowe et al. [36] showed that for variance components based QTL mapping methods, the test statistic and the variance explained can be hugely inflated if multiple testing and the underlying genetic architecture are not properly accounted for. Given that over 10 000 tests were performed, many of which were highly correlated due to the overlap of regions, and the novelty of the approach, we derived the empirical distribution of the test statistic using ACTA [34] to perform 100 permutations for each of the traits resulting in empirical thresholds for individual tests ranging from 17.6 for g_f_ to 18.8 for g_c_ for a type 1 error rate of 5%. As 100 permutations is not sufficient to ensure a stable estimate of the threshold, but testing 10,908 regions for three traits hundreds of times is computationally intensive, we repeated the analyses using non-overlapping windows and carried out a further 500 permutations. A permutation involved randomly permuting the phenotypic and genotypic data and testing 5454 alternate or non-over-lapping regions on the permuted data set. For each set of permuted data; i) regional population-sense heritabilities were estimated for all regions (each spanning 101 SNPs) and ii) The top ten regions ranked by LRT test statistic from each permutation were simultaneously fitted into a linear model to determine their combined contribution. These were fitted with and without a ‘polygenic’ component. This gave the empirical distribution of the test statistic under the null hypothesis for fitting a single region and for when the ten top ranking regions are fitted simultaneously.

### ‘One SNP at a time’ genome-wide association analysis

The software package PLINK [37] was used to carry out single SNP association tests to assess whether the SNPs of greatest significance were associated with the regions from [4] that explained the greatest amount of genetic variation.

## Results

### Variance captured by all autosomal SNPs or population-sense heritability

For simplicity we define the proportion of phenotypic variance captured by SNP genotypes in unrelated individuals as population-sense heritability (h^2^_ps_) to distinguish it from the often used narrow and broad sense heritability [29]. Heritabilities, phenotypic and genetic correlations are given in Table 1. Population-sense heritability estimates for cognitive traits ranged from 0.19 (s.e. 0.2) to 0.37 (s.e. 0.19). Estimates for crystallised intelligence are similar to those from the larger previous study [8]. Fluid intelligence estimates differ slightly due to differences in sample size, study design and population demographics. Fluid intelligence was highly genetically correlated to both cognitive change r_A_ = 0.95 (s.e. 0.25), and to crystallised intelligence r_A_ = 0.66 (s.e. 0.34) (i.e. the amount of correlation emerging from pleiotropy is high). There was little genetic correlation between crystallised intelligence and cognitive change r_A_ = 0.008 (s.e. 0.53).

**Table 1:** Population-sense heritability (diagonal), phenotypic (upper diagonal) and genetic (lower diagonal) correlations for measures of general intelligence and cognitive decline estimated from relationship matrices based on 547,750 SNP genotypes.

Trait	Crystallised Intelligence	Fluid Intelligence	Cognitive change
Crystallised intelligence (n = 1791)	0.36 (0.19)	0.59 (0.01)	0.22 (0.02)
Fluid intelligence (n = 1706)	0.66 (0.34)	0.19(0.20)	0.78 (0.009)
Cognitive change (n = 1602)	0.0084(0.53)	0.95 (0.25)	0.26(0.22)

Heritabilities on diagonal, genetic correlations below diagonal, phenotypic correlations above diagonal and standard errors given in brackets.

### Regional population-sense heritability

The distributions of regional population-sense heritability estimates for the three traits are similar. Most regions explain variance close to zero with 1.7 to 2.5% explaining greater than 1% of variation, 0.07 to 0.18% explaining greater than 2%, and only 0.02% explaining greater than 3%.

**Figure 1:**
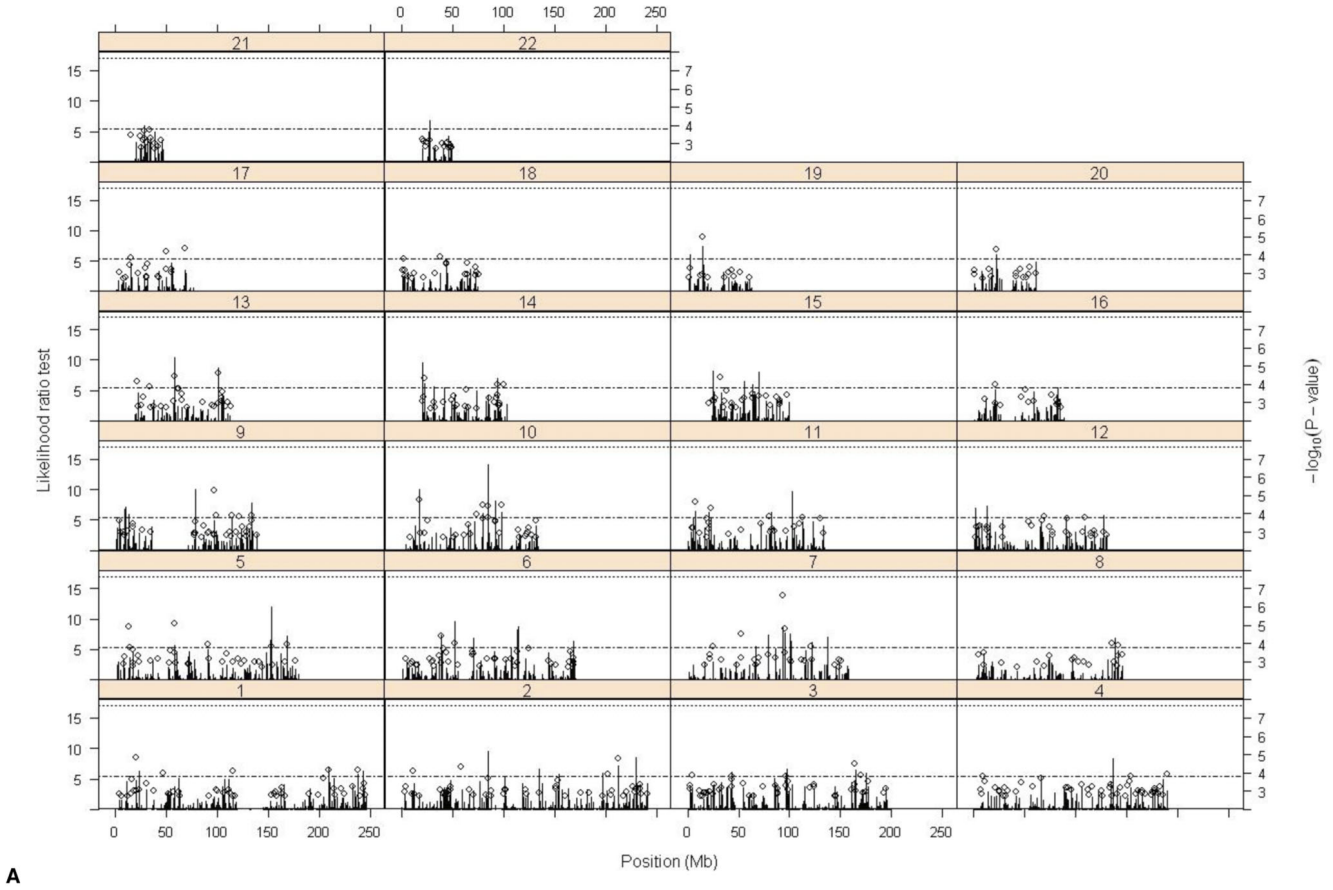
Plot of likelihood ratio test for phenotypic variance explained by each of 10,908 regions (groups of 101 consecutive SNPS) (bars) and −log_10_ P-values>2.7 for single SNP association (circles). Dashed line is 1% nominal significance threshold for LRT for individual regions, dotted line is 5% genome-wide significance threshold for individual regions obtained by permutation analysis. **A** crystallised intelligence n = 1791, **B** fluid intelligence n = 1706 , and **C** cognitive change n = 1602.

**Figure 2:**
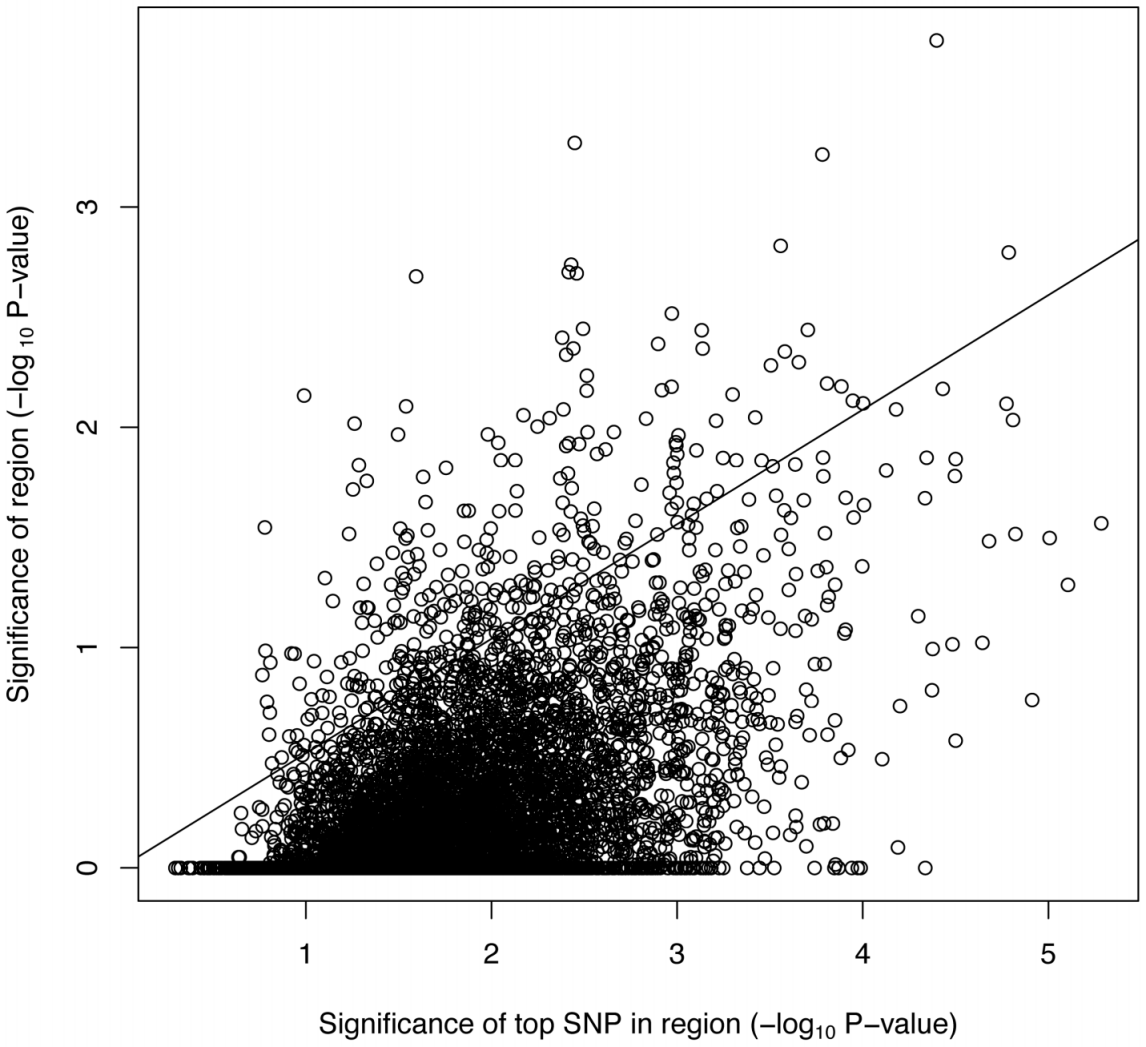
Comparison of significance of region and top SNP within region. Scatter plot of −log_10_ P-values for single SNP association of greatest significance in region and significance of LRT test for variance explained by entire region (each region contains 101 SNPs). Correlation coefficient is 0.52.

The likelihood ratio test statistic for the regional heritability scan across the genome and the most significant hits from the genome wide association analyses (−log_10_P-value>2.7) are given in Figure 1. Table 2 gives details of the top ten regions for each trait ranked by LRT and appendix 2 gives the known genes for each of these regions and pathway analysis. The top ten single SNP associations for the three traits were all within regions with h^2^_ps_>1% (Table S1 in File S1). The correlation between the greatest −log_10_ (*P*-value) for SNP association in each region and −log_10_ *P*-value from the LRT test for each region was 0.52 (Figure 2). When regions were ranked by LRT a region on chromosome 6 ranking 3^rd^ and 4^th^ for cognitive change and fluid intelligence respectively also contained the top SNP in the GWAS for cognitive change. For fluid intelligence, the top ranking region on chromosome 5 spanned the third ranking single SNP association (*P*<3.41E-06). This region on chromosome 5 associated with fluid intelligence was the only region for all three traits to exceed genome-wide significance at the *P*<0.10 threshold. When the top ten regions (Table 2) from each trait were fitted together in a LMM they explained 13% (P_perm_ = 0.58), 15% (P_perm_ = 0.11) and 18% (P_perm_ = 0.43) of the phenotypic variation for crystallised intelligence, fluid intelligence, and cognitive change respectively. Table 3 shows regions that explained greater than 1% of phenotypic variation in more than 1 trait including regions on chromosome 9 and 11 that potentially have pleiotropic effects on all three traits.

**Table 2:** Variance explained for top ten regions ranked by significance or LRT for crystallised and fluid intelligence and cognitive decline.

Chr	region start (bp)	region end (bp)	h^2^_ps_ region	s.e	h^2^_ps_ full model^a^	s.e.	LRT	Greatest single SNP association in region −LOG_10_ (P)	SNP Var (r^2^)
Crystallised Intelligence									
10	84493034	84943238	0.01	0.008	0.011^b^	0.007	14.08	4.4	0.009
5	153024650	153532086	0.02	0.01	0.017	0.009	12.07	2.45	0.003
10	84323605	84670475	0.02	0.012	-	-	11.85	3.78	0.008
13	57449351	58113705	0.01	0.008	0.01	0.006	10.48	4.45	0.008
9	78430995	78767837	0.01	0.008	0.008	0.006	10.08	3.56	0.008
10	17430161	17790975	0.01	0.008	0.015	0.008	9.95	4.79	0.008
11	102565882	102978790	0.02	0.01	0.015	0.008	9.72	2.43	0.005
14	20640453	21072443	0.03	0.014	0.02	0.012	9.57	2.42	0.004
6	51858157	52238923	0.01	0.008	0.007	0.006	9.55	2.46	0.005
2	84702898	85301342	0.01	0.008	0.013	0.008	9.49	1.59	0.003
13	100772901	101089435	0.02	0.009	0.014	0.008	8.78	2.97	0.002
Fluid Intelligence									
5	126711782	127335370	0.02	0.009	0.013	0.008	16.00	5.47	0.013
6	39140691	39378453	0.03	0.013	0.016	0.012	14.10	3.74	0.009
13	65117143	65633593	0.02	0.01	0.015	0.01	14.07	4.15	0.009
6	740414	1013400	0.02	0.009	0.013	0.008	12.36	4.74	0.011
6	39236400	39493104	0.04	0.018	-	-	12.34	3.52	0.008
11	102565882	102978790	0.02	0.009	0.015	0.008	11.55	4.42	0.010
9	78430995	78767837	0.01	0.009	0.01	0.007	11.07	3.25	0.007
11	102824059	103220693	0.01	0.007	-	-	10.91	3.34	0.007
3	101162780	101999012	0.02	0.011	0.02	0.011	10.55	5.04	0.012
5	33703559	34034521	0.02	0.012	0.016	0.01	9.44	0	0.005
2	151358558	151655394	0.02	0.01	0.012	0.008	9.37	3.43	0.008
5	127010643	127650653	0.01	0.009	0.015	0.008	9.33	0.91	0.007
Cognitive Change									
4	53606097	54158143	0.02	0.009	0.01	0.008	10.44	4.4	0.011
15	90960003	91404141	0.02	0.011	0.017	0.011	10.15	4.83	0.012
6	740414	1013400	0.02	0.009	0.014	0.009	10.08	5.57	0.014
4	62441864	63300488	0.03	0.014	0.024	0.012	9.50	2.57	0.006
6	891665	1138987	0.02	0.009	-	-	8.77	1.56	0.003
6	12418779	12930959	0.02	0.009	0.014	0.009	8.70	4.02	0.010
2	237734083	238123037	0.02	0.011	0.016	0.006	8.30	3.6	0.009
13	98189341	98677491	0.04	0.022	0.035	0.018	8.12	2.59	0.006
14	64270578	64666246	0.02	0.011	0.016	0.01	8.10	2.77	0.006
6	88043140	88678348	0.01	0.007	0.008	0.007	8.08	3.37	0.008
4	148617678	149254898	0.02	0.01	0.023	0.013	7.92	2.71	0.006

^a^ heritability of region when full model fitting 11 variance components first ten independent (i.e. non overlapping) regions and rest of genome.

^b^ Only the best supported of multiple overlapping regions was fitted.

**Table 3:** Pleiotropic regions affecting multiple traits.

Chr	region start (bp)	region end (bp)	h^2^ Crystallised	s.e.	h^2^ Fluid	s.e.	h^2^ Cog change	s.e.
6	740414	1013400	0.00	0.01	0.02	0.01	0.01	0.01
14	64270578	64666246	0.00	0.00	0.02	0.01	0.02	0.01
9	78430995	78767837	0.01	0.01	0.01	0.01	0.02	0.00
10	17430161	17790975	0.01	0.01	0.02	0.00	0.00	0.01
11	102565882	102978790	0.02	0.01	0.02	0.01	0.01	0.01
11	102824059	103220693	0.02	0.01	0.02	0.01	0.01	0.01

Regions were defined by number of SNPs; hence there was variation in physical length of regions across the genome, with the average region spanning 534 kb. No relationship was found between the physical length of a region and its significance or the amount of additive genetic variation explained (Figure S1 in File S1).

### Permutation analyses

**Figure 3:**
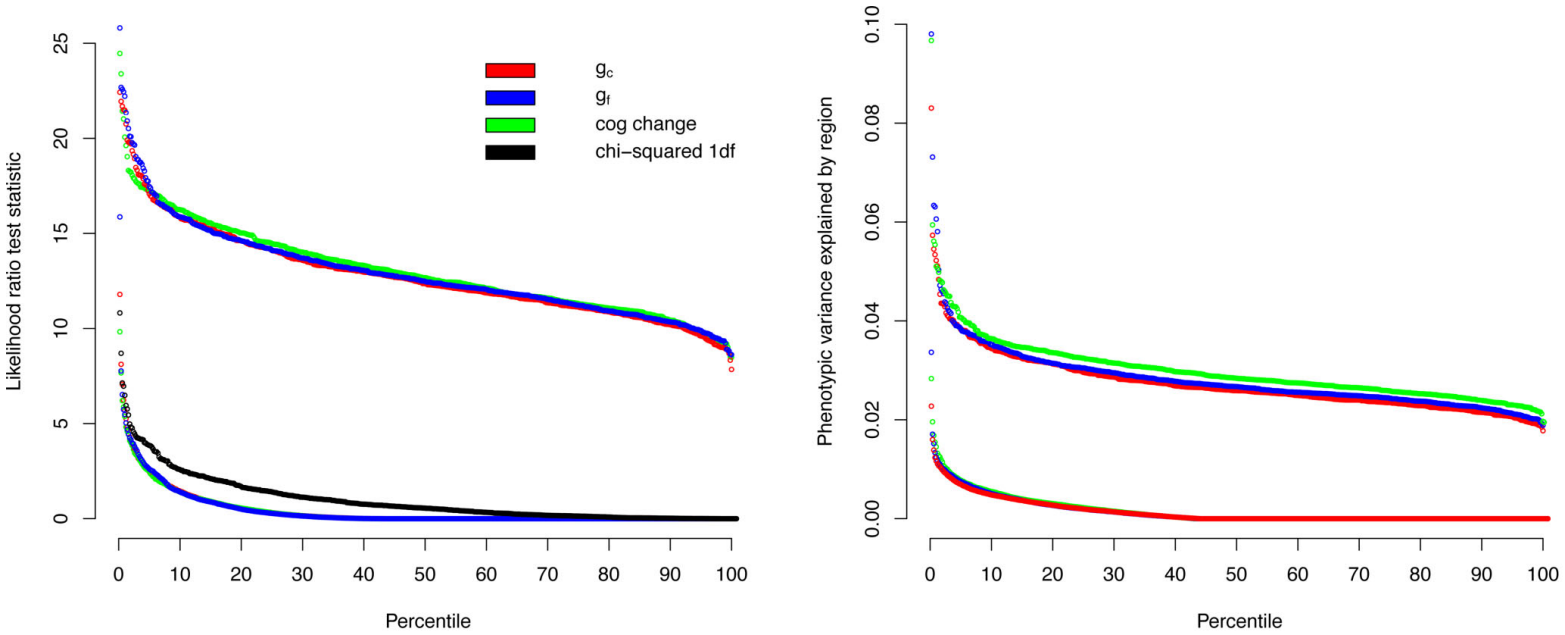
Distribution of the likelihood ratio test and variance explained under the null hypothesis. Comparison of the distribution of likelihood ratio test and variance explained for 5454 regions spanning 101 SNPs for fluid intelligence, crystallised intelligence and cognitive change. Lower set of distributions for each plot are from the real data, upper set are the 5% genome-wide significance threshold from each of 500 permuted data sets i.e. empirical null distribution.

To estimate empirical thresholds, phenotypic data for each of the three traits were permuted 500 times to attain an estimate of the null distribution when genotype and phenotype were randomly assorted. We performed 5,454 REML analyses across the genome for each of the permuted data sets resulting in over 8.2 million single tests. The results were ranked by log likelihood and compared to a null model using an LRT. The resulting genome-wide significance thresholds for the LRT (*P*<0.05) were 17.2 for crystallised intelligence, 17.5 for fluid intelligence and 17.08 for cognitive change Figure 3 shows that the distributions of the test statistic for the three traits were very similar and that they were highly inflated when compared to the expectation of the null distribution for a single test. Thresholds were close to those for the 10,908 tests but less conservative than a Bonferroni correction for 5,454 independent tests which would result in a 5% threshold of 19.7. Table 4 shows that the genome-wide threshold values were stable after 300 permutations indicating that 500 permutations was sufficient to estimate 5 and 10% genome-wide thresholds.

**Table 4:** Genome wide thresholds for the Likelihood Ratio Test (LRT) derived from N permutations.

Genome-wide threshold for LRT						
	Fluid intelligence		Crystallised intelligence		Cognitive Change	
N	*P*<0.05	*P*<0.10	*P*<0.05	*P*<0.10	*P*<0.05	*P*<0.10
100	19.0	16.5	19.4	16.3	18.0	16.6
200	17.8	15.8	18.1	16.3	17.6	16.5
300	17.5	15.8	17.6	16	17.0	16.1
400	17.4	15.8	17.1	15.8	17.3	16.3
500	17.5	15.8	17.1	15.9	17.2	16.2

The distributions from the permutation analysis (Figure 3) show that by chance in 5% of cases the variance explained by a region exceeded 3.8, 3.8 and 4.0% for g_c_, g_f_ and cognitive change respectively.

For each permutation the top ten regions were identified, i.e. those with the greatest likelihoods and fitted simultaneously into a LMM. An LRT was calculated as twice the difference between the log likelihood of a model fitting ten regions and a null model without a genetic effect, and we did not fit a polygenic model when testing the top ten regions. The 95^th^ percentile was used to estimate a 5% genome-wide threshold for significance of the LRT between a model fitting the top ten regions of the genome; and a null model. The polygenic component was omitted as the original genetic structure was removed by the permutation of genotypes and phenotypes. The 5% genome-wide threshold was P<3.3E-24 for crystallised intelligence, P<1.42E-24 for fluid intelligence and P<1.03 E-24.

### Functional population-sense heritability

**Figure 4:**
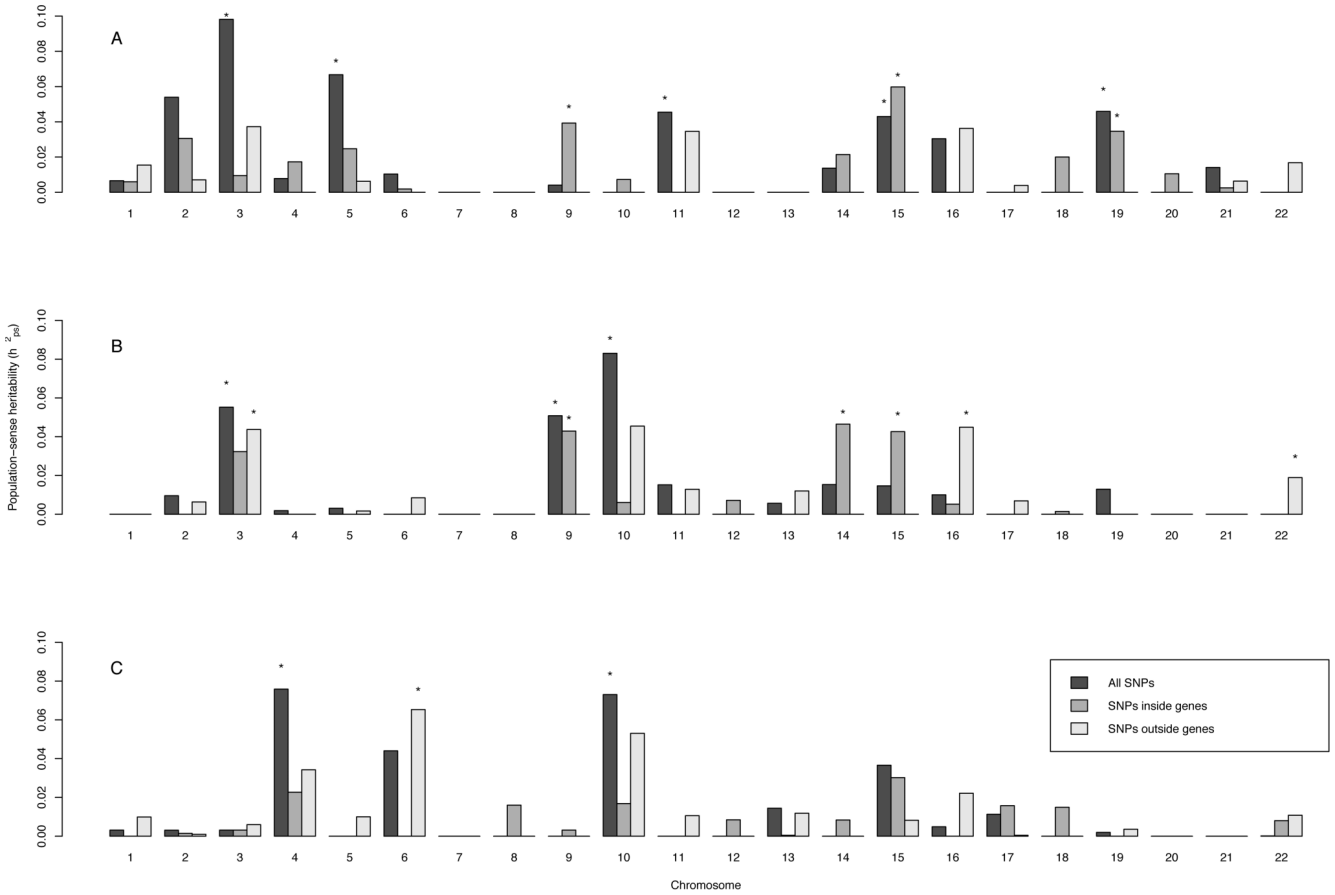
Distribution of population sense-heritability inside and outside genes. Distribution of heritability estimated from all SNPs, SNPs inside genes and SNPs outside genes by chromosome for crystallised intelligence, fluid intelligence and cognitive decline.

Figure 4 shows estimates of population-sense heritability for each of the 22 autosomes, and for h^2^_ps_ estimates using information from SNPs inside genes and estimates using information from SNPs outside genes for each chromosome and trait. For crystallised intelligence heritability estimates from SNPs on autosomes 3, 5, 11, 15 and 19 were significantly different from zero. When divided further chromosomes 9, 15 and 19 had significant estimates for h^2^_ps_ within genes. For fluid intelligence, estimates of h^2^_ps_ on chromosomes 3, 9 and 10 were significant, explaining 6, 5, and 8% phenotypic variance, respectively. Autosomal h^2^_ps_ within genes was significant for chromosomes 9, 14 and 15 and outside genes for chromosomes 3, 16 and 22. For cognitive change chromosomes 4 and 10 had significant estimates of h^2^_ps_ with chromosome 6 significant for h^2^_ps_ outside genes.

Genetic variation of the traits differed across autosomes and for SNPs within or outside genes. SNPs within genes explained 48, 64 and 38% of the total genetic variation for *g*_c_, *g*_f_ and cognitive change respectively. There was no correlation between estimates of autosomal heritability and the number of SNPs used to estimate each genetic relationship matrix (Table S2 in File S1). Distributions of allele frequencies for SNPs inside and outside genes did not differ *P*<0.99).

### Brain-related intermediate traits

The top region associated with g_f_ was genome-wide significant at the 10%, however, even if the locus was truly associated with g_f_ we were not expecting a high level of statistical support due to the small sample size of the study. In order to gather further independent evidence that could support or reject the association of the locus with g_f_ we assembled previously published data of brain-measured intermediate phenotypes [38]. Within the chromosome 5 region we found two DNAm sites, cg04431054 and cg15851800 and two mRNA probes ILMN_1652306 and ILMN_1685140. DNAm sites cg04431054 and cg15851800 are located 381 base-pairs apart, cg04431054 is 277 base-pairs upstream of PRRC1, and cg15851800 is 104 base-pairs downstream of the transcription start site of PRRC1, which spans chromosome 5 at base-pair location 126,853,301–126,890,781. ILMN_1685140 targets transcripts of PRRC1 (Proline-Rich Coiled-Coil 1) and ILMN_1652306 transcripts of MEGF10, a receptor for amyloid beta uptake, located between position 126,626,523 and 126,801,429. All four intermediate phenotypes were measured on tissue from the Cerebellum (CRBL), Frontal Cortex (FCTX), the Pons (PONS) and the Temporal Cortex (TCTX). ILMN_1652306 did not pass our quality control procedure for the CRBL and thus was excluded from further analyses. Regional genetic relationships were estimated from 86 available SNPs located within the top 101 SNPs region associated with g_f_. h^2^_ps_ was estimated with ACTA [34].

The 86 SNPs located on chromosome 5 between 126711782–127335370 base-pairs explain a significant (P<0.0001) proportion of the phenotypic variation of cg04431054 for each of the four brain tissues (Table 5). h^2^_ps_ of cg04431054 measured in the CRBL, FCTX, PONS and TCTX brain regions was 0.46, 0.24, 0.28 and 0.33, respectively.

**Table 5:** Population-sense regional heritability for each brain-measured intermediate phenotype within the top g_f_ associated region on chromosome 5.

		Regional heritability of brain-measured intermediate traits		
Intermediate Phenotype	Tissue	h^2^_ps_	SE	P
*cg04431054*	*CRBL*	*0.463*	*0.124*	*1.370E-08*
cg15851800	CRBL	0.000	0.075	0.500
*cg04431054*	*FCTX*	*0.237*	*0.104*	*1.190E-05*
cg15851800	FCTX	0.020	0.050	0.325
*cg04431054*	*PONS*	*0.278*	*0.111*	*1.270E-05*
cg15851800	PONS	0.003	0.046	0.477
*cg04431054*	*TCTX*	*0.326*	*0.110*	*1.020E-08*
cg15851800	TCTX	0.082	0.078	0.063
ILMN_1685140	CRBL	0.025	0.053	0.315
ILMN_1652306	FCTX	0.000	0.049	0.500
ILMN_1685140	FCTX	0.000	0.041	0.500
ILMN_1652306	PONS	0.000	0.033	0.500
ILMN_1685140	PONS	0.046	0.051	0.075
ILMN_1652306	TCTX	0.000	0.079	0.500
ILMN_1685140	TCTX	0.000	0.045	0.500

Tissue: brain region, h^2^_ps_: estimated regional population-sense heritability, SE: estimated standard error of the regional population-sense heritability. P:p-value from the LRT test testing the significance of the genetic variance component.

So far, we have shown that the 623 kb region of chromosome 5 associated with g_f_ is associated with cg04431054 levels in the CRBL, FCTX, PONS and TCTX brain regions. However, we have not yet shown a direct link between cg04431054 levels and g_f_. To do that, we estimate the effect of the 86 SNPs on the brain phenotypes and construct a genetic score [39] for each individual with g_f_ phenotypes. A significant regression of genetic score for cg04431054 with g_f_ would indicate a link between the levels of cg04431054 and g_f_. Only one of the four brain regions (TCTX) showed a significant association with g_f_ (P = 0.004), and explained 0.5% of the phenotypic variance. The regression coefficient was positive (0.295, se = 0.004) indicating a positive correlation between methylation levels and g_f_. Hence, our analyses of brain-related intermediate phenotypes provides supporting evidence of the region being truly associated with g_f_, uncovers the likely target region of the brain associated with g_f_ and identifies PRRC1 as a candidate gene for g_f_.

## Discussion

We implemented a recently proposed method of genome scanning by expanding single SNP analysis to the estimation of genetic variance explained by regions spanning 101 co-located SNPs. After deriving empirical thresholds by permutation analysis we show that stringent thresholds close to that of a bonferroni correction are necessary for evaluating the likelihood ratio test statistic and that the distribution of multiple tests is highly inflated when compared to the null distribution for a single test. This is also true for estimates of heritability (h^2^_ps_). Table 2 shows that within the top ten regions ranked by LRT, only a region on chromosome 6 for fluid intelligence and a region on chromosome 13 for cognitive change explained a greater proportion of the genetic variance (h^2^_ps_) than 95% of the ranked permutation analyses. Despite this the LRT for the comparison of the linear models did not achieve genome wide significance for either of these regions.

**Figure 5:**
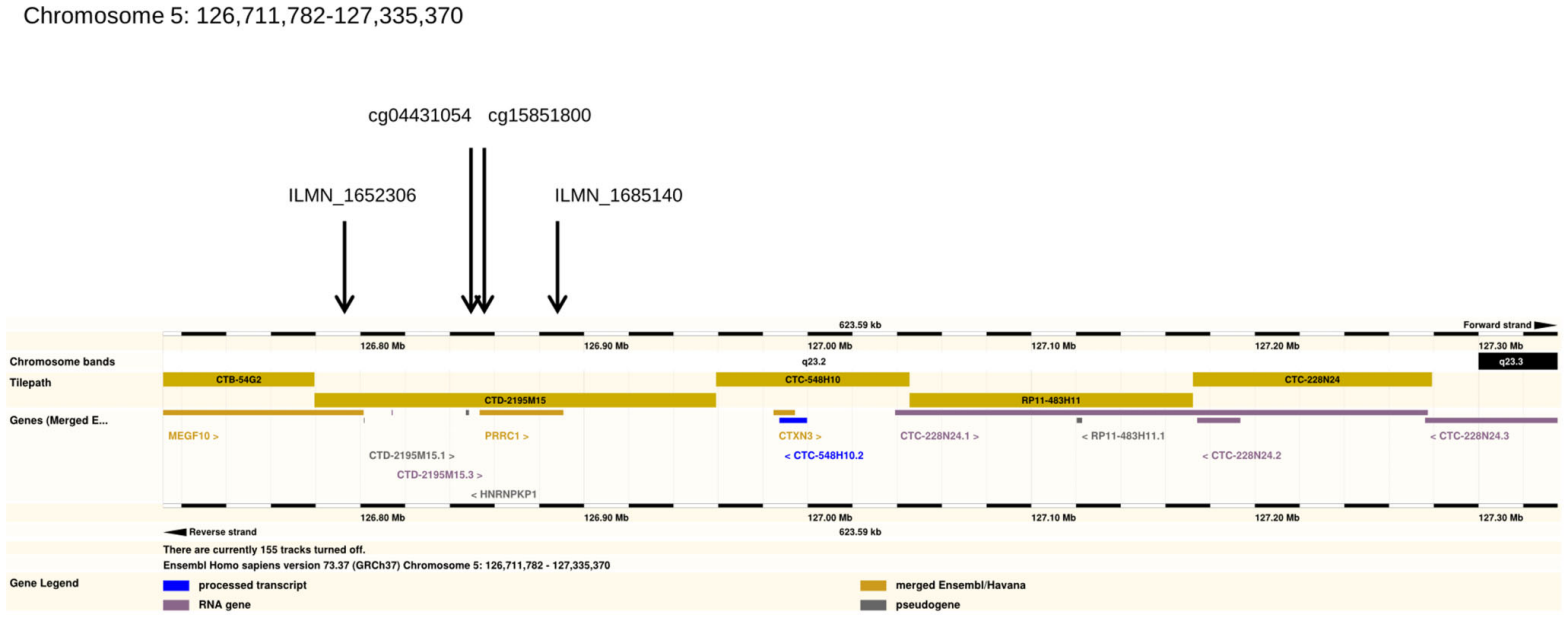
Region on chromosome 5 significantly associated with fluid intelligence. Annotation from Ensembl genome browser.

We did find a genome wide significant region (P<0.10) for the LRT statistic on chromosome 5 associated with fluid intelligence. The region spans the *CTXN3* gene (*cortexin 3*) (Figure 5), a brain (including foetal brain) and kidney specific integral membrane protein, highly enriched in cortex and located on 5q23. This gene has been previously identified as a candidate for schizophrenia and measures of cognitive change [40]. In the GWAS, the third highest ranking SNP *rs790837* (P<10^−6^) is located at position 127004506, 10 kb away from this gene.

The *CTXN3-SLC12A2* region is a strong candidate region and has been linked to brain function and schizophrenia in multiple studies [41], [42], [43]. The relationship between pre-morbid measures of intelligence and the risk of schizophrenia is also documented as greater than with many other psychoses [44], [45]. Although the overlapping region containing *SLC12A2* ranked within the top ten regions with an LRT of 9.33 (Table 2), here the region containing *cortexin 3* (LRT = 16) was the only region to achieve genome wide significance (P<0.10). Nonetheless, the strongest evidence suggests that variation of methylation levels at the promoter region of PRRC1 are mediating variation if g_f_. The function of the Golgi-associated PRRC1 gene in the brain is unknown and will require follow-up functional studies.

The population-sense heritabilities for fluid intelligence are lower than those previously reported possibly due to an older demographic. Family based (narrow-sense) estimates of heritability for IQ related traits have been shown to decline somewhat with age [46]. This, in part, will be due to an increase in environmental variance.

### Autosomal heritability

In general the estimates of genomic heritability for chromosomes reflected the analysis of smaller regions in that the regions with the highest test statistics are located on chromosomes explaining the greatest variance. The sum of heritability estimates for individual chromosomes was inflated by 20–50% compared to estimating the heritability for the entire genome. When heritabilities were estimated from SNPs inside and outside genes (i.e. fitting 44 variance components) heritability for fluid intelligence was doubled when compared to fitting the 22 autosomes (Table S3 in File S1). This could be due to fitting so many correlated variance components simultaneously; however, estimates for crystallised intelligence remained stable. It is also possible that this is due to the lack of independence of SNPs within chromosomes inflating estimates, although fitting all 44 variance components simultaneously should account for this. It is probable that more information from a greater number of individuals would enable more precise estimates of covariances and therefore more accurate estimation and partition of variance components. It is also possible that crystallised intelligence is a more polygenic trait with some genetic variance contributed from most chromosomes whereas fluid intelligence and cognitive change show variation around many autosomal estimates which are truly zero.

### Pleiotropy

Only 2.5% of regions show an h^2^_ps_ greater than 1%. Despite this there is much overlap between the three traits with top regions affecting multiple traits (Table 3). This suggests that the three traits are likely to be affected by the same genes and biological pathways. However, the direction of the effects in these regions will tend to be different for traits such as cognitive change and crystallised intelligence that show a genetic correlation close to zero (Table 1). A single region might also contain linked QTL alleles or regulatory factors in coupling or cis.

It is also feasible that regional significance is biased by other factors making a region more or less likely to explain variation in one or multiple traits. We found no relationship between physical length of region and test statistic. Yang et al. [29] proposed that the genetic variation explained by a region was proportional to the total length of genes. We did not find this in the current study. The unadjusted r^2^ values for the relationship between heritability of autosomes and total length of known genes on each chromosome was 0.14, 0.02, and 0.01 for crystallised intelligence, fluid intelligence, and cognitive change with corresponding p-values of 0.07, 0.54, and 0.65. It is possible that this is dependent on the heritability and the genetic architecture of the trait, i.e. the more polygenic the trait the higher the correlation between the amount of heritable genetic material on each chromosome and the estimate of heritability. This is reflected in Yang et al.'s report where, although height and BMI were highly correlated with the length of genes, there was variation amongst traits with an r^2^ value of only 0.02 for von Willebrand factor.

Distributions of the regional heritability test statistic (−log_10_ P-value) were compared across traits and gender using a Kolmogorov-Smirnov test. Cognitive change differed from fluid and crystallised intelligence (P<2.2E-16 and P<5.0E-11, respectively). Differences between crystallised and fluid intelligence were less marked (P<0.01). Interestingly, we found some evidence that the distribution of heritability across the genome for cognitive change differs in males and females. Genetic variation was higher in females and the Kolmogorov-Smirnov test of the distributions of heritabilities for the 10,908 regions in males (n = 871) and females (n = 933) was suggestive at *P*-value of 0.06, although the test does not account for the correlation of the regions and is likely to be inflated. A previous study showed higher variation within males for a measure of general intelligence [47]. It is possible that the increased environmental variance attributable to old age happens sooner in males than females.

It is not clear from this study whether there is utility in a method which expands single SNP analyses to encompass genomic regions and that it is able to capture complex local genetic architectures. We acknowledge the limitations of our analysis. Statistical power and accuracy of estimation of variance components is most certainly an issue. Fluid intelligence and cognitive change are important traits and to date lifetime measurements are rare. This limits our ability to increase the sample size. We have shown that the heritability of a region or autosome is not merely a function of its length or the number of genes contained therein. It will be desirable to test the methodology with much larger data sets. It would be interesting to assess whether the regions of greatest significance are enriched for psychiatric genes in comparison to randomly selected regions. Gene set enrichment analyses developed for microarray analysis could be a useful tool for this.

## Conclusions

Using a recently proposed population-based linkage scan of the genome we have conducted a search for regions significantly associated with measures of cognition and age related cognitive change. Permutation analysis shows that test statistics and variance explained by a single window were highly inflated when compared to the assumption of a chi square distribution for a single test. We found a significant region on chromosome 5 associated with fluid intelligence explaining 2% of phenotypic variation.

Although single SNP and regional analysis have similar profiles, the ranking of the top regions differ. The regions with the highest test statistic although not genome-wide significant did affect multiple traits and encompass biologically plausible and interesting putative candidate genes. These regions indicate areas of the genome where re-sequencing efforts could be focused to disentangle the fine scale contribution of linked genes and pathways. Although our methodology would benefit from larger sample sizes and increased power, the results give new insights into the study of general intelligence and the underlying mechanisms of cognitive change.

## Supporting Information

File S1 **This file contains Figure S1 and Tables S1–S3.** **Figure S1**, Plot of relationship between length of region and LRT. **Table S1**, Top Results for single SNP association. **Table S2**, Correlations between autosomal estimates of h^2^_ps_ for Crystallised intelligence, Fluid intelligence and Cognitive change with the number of SNPs used to estimate GRM, the number of genes and total length of genes and heritability of autosomes. **Table S3**, Heritability estimates for the 22 autosomes.
(DOCX)
