# Correction: Complex Variation in Measures of General Intelligence and Cognitive Change

**DOI:** 10.1371/journal.pone.0091622

**Published:** 2014-03-25

**Authors:** The PLOS ONE Staff

Figure 1 is missing panels B and C. The authors have provided a corrected version here. The publisher apologizes for the error.

**Figure 1:**
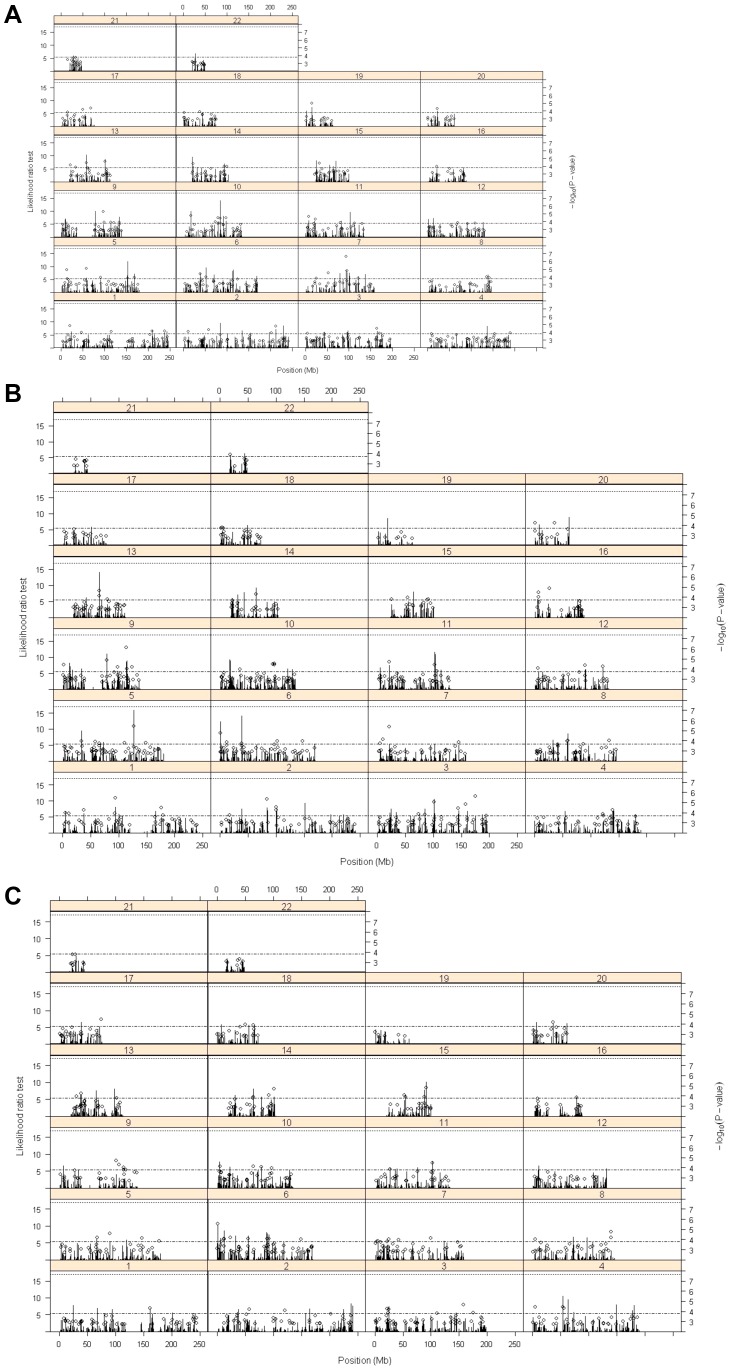
Plot of likelihood ratio test for phenotypic variance explained by each of 10,908 regions (groups of 101 consecutive SNPS) (bars) and −log_10_ P-values>2.7 for single SNP association (circles). Dashed line is 1% nominal significance threshold for LRT for individual regions, dotted line is 5% genome-wide significance threshold for individual regions obtained by permutation analysis. A crystallised intelligence n  =  1791, B fluid intelligence n  =  1706, and C cognitive change n  =  1602.
