# Supplementary material for: Complex Variation in Measures of General Intelligence and Cognitive Change
Source: PLoS One. 2013 Dec 12;8(12):e81189. doi: 10.1371/journal.pone.0081189 (PMC3865348; doi:10.1371/journal.pone.0081189)
Supplement: File S1 — This file contains Figure S1 and Tables S1–S3. Figure S1, Plot of relationship between length of region and LRT. Table S1, Top Results for single SNP association. Table S2, Correlations between autosomal estimates of h2 ps for Crystallised intelligence, Fluid intelligence and Cognitive change with the number of SNPs used to estimate GRM, the number of genes and total length of genes and heritability of autosomes. Table S3, Heritability estimates for the 22 autosomes. (DOCX) [file pone.0081189.s001.docx]

**Supplemental Data**

**Contains 1 figure and 3 tables**

1. **Figure S1.** Plot of relationship between length of region and LRT.
2. **Table S1.** Top Results for single SNP association.
3. **Table S2.** Correlations between autosomal estimates of h^2^_ps_ for Crystallised intelligence, Fluid intelligence and Cognitive change with the number of SNPs used to estimate GRM, the number of genes and total length of genes and heritability of autosomes.
4. **Table S3.** Heritability estimates for the 22 autosomes.

**
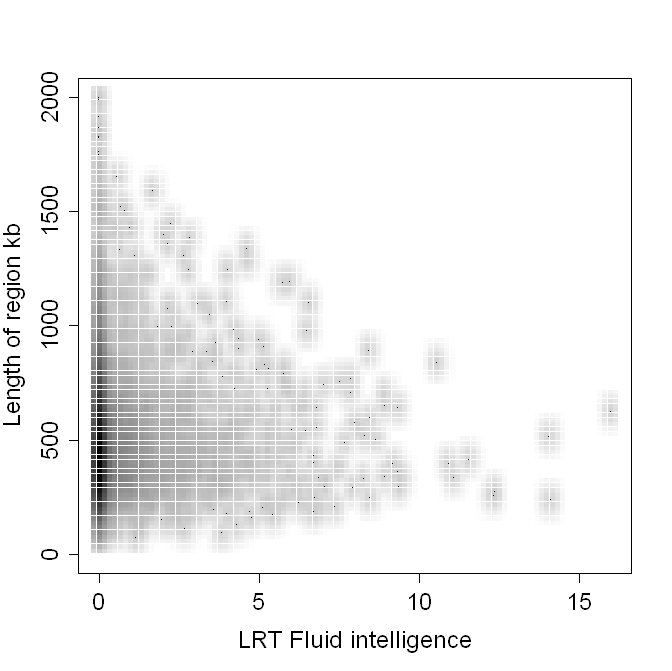
**

**Figure S1.** Scatter Plot of likelihood ratio test (LRT) statistic for fluid intelligence against physical length of region. Intensity of colour denotes density therefore regions are most likely to have an LRT of zero and approximately 500kb in length.

|  |  |  |  |  |  |  |
| --- | --- | --- | --- | --- | --- | --- |
|  |  |  |  |  |  |  |
|  |  |  |  |  |  |  |
|  |  |  |  |  |  |  |
|  |  |  |  |  |  |  |
|  |  |  |  |  |  |  |
|  |  |  |  |  |  |  |
|  |  |  |  |  |  |  |
|  |  |  |  |  |  |  |
|  |  |  |  |  |  |  |
|  |  |  |  |  |  |  |
|  |  |  |  |  |  |  |
|  |  |  |  |  |  |  |

**Table S1 Top Results for single SNP association**

| SNP | Chr | *P*-value | Position (bp) | h^2^ region | s.e. |
| --- | --- | --- | --- | --- | --- |
| Crystallised Intelligence | | | | | |
| rs7782469 | 7 | 2.47E-07 | 93761080 | 0.01 | 0.009 |
| rs2052213 | 7 | 4.89E-07 | 93703633 | 0.01 | 0.009 |
| rs1861114 | 7 | 1.53E-06 | 93721535 | 0.01 | 0.009 |
| rs12686467 | 9 | 5.19E-06 | 97471458 | 0.01 | 0.01 |
| rs41542 | 7 | 7.16E-06 | 93559972 | 0.01 | 0.009 |
| rs158281 | 5 | 7.85E-06 | 57760549 | 0.01 | 0.007 |
| rs12461006 | 19 | 9.85E-06 | 14922216 | 0.01 | 0.007 |
| rs2401793 | 5 | 1.22E-05 | 13385878 | 0.01 | 0.006 |
| rs10047178 | 1 | 1.51E-05 | 20643873 | 0.01 | 0.009 |
| rs2188505 | 7 | 1.55E-05 | 95374205 | 0.01 | 0.01 |
| Fluid Intelligence | | | | | |
| rs10817343 | 9 | 7.54E-07 | 1.14E+08 | 0.01 | 0.009 |
| rs6445137 | 3 | 2.52E-06 | 1.75E+08 | 0.02 | 0.01 |
| rs790837 | 5 | 3.41E-06 | 1.27E+08 | 0.02 | 0.009 |
| rs3747965 | 1 | 3.41E-06 | 94115152 | 0.01 | 0.006 |
| rs12700284 | 7 | 4.09E-06 | 21413414 | 0.01 | 0.008 |
| rs1902156 | 2 | 4.52E-06 | 84431095 | 0.01 | 0.006 |
| rs9864288 | 3 | 9.09E-06 | 1.02E+08 | 0.02 | 0.011 |
| rs10981396 | 9 | 1.31E-05 | 1.14E+08 | 0.01 | 0.009 |
| rs11145045 | 9 | 1.40E-05 | 78557187 | 0.01 | 0.007 |
| rs4787912 | 16 | 1.45E-05 | 26994169 | 0.01 | 0.007 |
| Cognitive Decline | | | | | |
| rs1764142 | 6 | 2.69E-06 | 956570 | 0.02 | 0.009 |
| rs7738087 | 6 | 2.99E-06 | 939790 | 0.02 | 0.009 |
| rs845869 | 6 | 1.12E-05 | 943062 | 0.02 | 0.009 |
| rs10520716 | 15 | 1.49E-05 | 91292021 | 0.02 | 0.011 |
| rs7831232 | 8 | 1.82E-05 | 1.40E+08 | 0.00 | 0.006 |
| rs1004904 | 14 | 2.05E-05 | 1.02E+08 | 0.02 | 0.011 |
| rs4812661 | 20 | 2.11E-05 | 41008537 | - | - |
| rs182762 | 9 | 2.14E-05 | 1.01E+08 | 0.01 | 0.006 |
| rs10936058 | 3 | 2.32E-05 | 1.58E+08 | 0.01 | 0.009 |
| rs4916794 | 5 | 2.71E-05 | 89609684 | 0.01 | 0.008 |

**Table S2 Correlations between autosomal estimates of h^2^_ps_ for Crystallised intelligence, Fluid intelligence and Cognitive change with the number of SNPs used to estimate the GRM, the number of genes and total length of genes.**

|  | Crystallised Intelligence | Fluid Intelligence | Cognitive change |
| --- | --- | --- | --- |
|  | r^2^ h^2^_ps_  (P-val) | r^2^ h^2^_ps_  (P-val) | r^2^ h^2^_ps_  (P-val) |
| # of genes on chr | 0.06 (0.26) | 0.006(0.72) | 0.0001(0.99) |
| total length of genes on chr | 0.15(0.07) | 0.02(0.54) | 0.01(0.65) |
| total # of SNPs | 0.03(0.20) | 0.02(0.43) | 0.001(0.34) |
| # SNPs within Genes | 0.04(0.19) | 0.009(0.38) | 0.03(0.57) |
| # SNPs outwith Genes | 0.02 (0.50) | 0.04(0.71) | 0.01(0.41) |

Undjusted r-squared values for relationship between estimates of heritability for individual chromosomes and total number of genes on each chromosome or total length of genes on each chromosome (bp). Information source Ensembl build 37.

**Table S3 Genomic Heritability estimates for Crystallised intelligence, Fluid Intelligence and Cognitive change for 22 autosomes using 547 950 SNP genotypes**

| Chr | h^2^ *G*_c_ | s.e. | h^2^ *G*_f_ | s.e. | h^2^ Cog change | s.e. |
| --- | --- | --- | --- | --- | --- | --- |
| 1 | 0.01 | 0.056 | 0 | 0.063 | 0 | 0.066 |
| 2 | 0.05 | 0.056 | 0.01 | 0.061 | 0 | 0.061 |
| 3 | 0.10* | 0.052 | 0.06 | 0.057 | 0 | 0.056 |
| 4 | 0.01 | 0.049 | 0 | 0.054 | 0.08* | 0.058 |
| 5 | 0.07* | 0.052 | 0 | 0.054 | 0 | 0.055 |
| 6 | 0.01 | 0.048 | 0 | 0.053 | 0.04 | 0.056 |
| 7 | 0 | 0.046 | 0 | 0.051 | 0 | 0.051 |
| 8 | 0 | 0.044 | 0 | 0.051 | 0 | 0.053 |
| 9 | 0 | 0.042 | 0.05* | 0.047 | 0 | 0.048 |
| 10 | 0 | 0.045 | 0.08* | 0.053 | 0.07* | 0.055 |
| 11 | 0.05* | 0.044 | 0.02 | 0.048 | 0 | 0.048 |
| 12 | 0 | 0.045 | 0 | 0.05 | 0 | 0.052 |
| 13 | 0 | 0.04 | 0.01 | 0.043 | 0.01 | 0.045 |
| 14 | 0.01 | 0.037 | 0.02 | 0.042 | 0 | 0.044 |
| 15 | 0.04* | 0.036 | 0.01 | 0.038 | 0.04 | 0.042 |
| 16 | 0.03 | 0.04 | 0.01 | 0.042 | 0 | 0.043 |
| 17 | 0 | 0.036 | 0 | 0.04 | 0.01 | 0.041 |
| 18 | 0 | 0.036 | 0 | 0.041 | 0 | 0.041 |
| 19 | 0.05* | 0.034 | 0.01 | 0.035 | 0 | 0.037 |
| 20 | 0 | 0.036 | 0 | 0.04 | 0 | 0.041 |
| 21 | 0.01 | 0.029 | 0 | 0.031 | 0 | 0.032 |
| 22 | 0 | 0.027 | 0 | 0.028 | 0 | 0.028 |
| Total | 0.44 |  | 0.28 |  | 0.27 |  |

**Appendixes**

**Appendix 1 Construction of cognitive phenotypes**

For each of the cohorts we constructed cognitive phenotypes of fluid-type and crystallized-type intelligence. Here, to represent crystallized intelligence (*g*_c_), we used: the National Adult Reading test.

For fluid-type intelligence, principal components analyses (PCA) were used to derive a general intelligence factor. In each case, the scores on a number of fluid-type cognitive tests were subjected to PCA. The tests used to form the *g*_f_ factor in the LBC1921 were the Moray House Test[[1](#_ENREF_1),[2](#_ENREF_2)], Raven’s Matrices[[3](#_ENREF_3)], Logical Memory[[4](#_ENREF_4)], and Verbal Fluency [[5](#_ENREF_5)]. For the LBC1936 the six tests from the WAIS-IIIUK[[6](#_ENREF_6)]were used. The ABC1936 g_f_ factor included the Moray House Test[[7](#_ENREF_7),[8](#_ENREF_8)], Raven’s Progressive Matrices[[3](#_ENREF_3)], Digit Symbol[[9](#_ENREF_9)], Uses of Common Objects[[5](#_ENREF_5)], and AVLT[[5](#_ENREF_5)]. In all cases a single component was indicated and was extracted. Thus, individuals’ scores on the first unrotated principal component were used as the indicator of general fluid-type intelligence (*g*_f_). All of the phenotypes were corrected for age and gender and the standardized residuals were used for all subsequent analyses.

*Cognitive change*

The *g*_f_ factors for each cohort were adjusted for prior cognitive ability using the Moray House Test scores at age 11, thus providing a quantitative measure of cognitive change from age 11 to old age. Both g_f_ and age 11 Moray House Test scores were adjusted for age in days at time of testing prior to the creation of the cognitive change measure. These measures were extracted and standardized independently for males and females.

**Appendix 2. Pathway analysis**

**Table A2 Genes located in top ten regions ranked by significance (LRT) for crystallised and fluid intelligence and cognitive change.**

Crystallised Intelligence

| Chr | Region start (bp) | Region end  (bp) | Known genes in region |
| --- | --- | --- | --- |
| 10 | 84493034 | 84943238 | *NRG3, U6* |
| 5 | 153024650 | 153532086 | *GRIA1, AC091960.1, AC091962.3.1, RP11-461L18.1.1, FAM114A2, MFAP3, AC010295.1* |
| 10 | 84323605 | 84670475 | *NRG3* |
| 13 | 57449351 | 58113705 | *RN7SKP6, PRR20A, PRR20B, PRR20C, PRR20D, PRR20E, MTCO2P3, SLC25A5P4, RPL31P53* |
| 9 | 78430995 | 78767837 | *PCSK5, AL359253.1* |
| 10 | 17430161 | 17790975 | *ST8SIA6 RP11-414K1.3.1 Y_RNA, RP11-390B4.4.1, PTPLA, RP11-390B4.5.1, STAM, snoU40, RP11-390B4.3.1* |
| 11 | 102565882 | 102978790 | *MMP27, MMP8, RP11-725K16.4.1, AP000619.5.1, AP000647.3.1, MMP10, MMP1, AP000619.6.1, MMP3, MMP12, RP11-725K16.2.1, U7, MMP13 RP11-690D19.1.1, RP11-690D19.3.1, DCUN1D5, DYNC2H1* |
| 14 | 20640453 | 21072443 | *20640453, 21072443, RP11-98N22.6.1, OR11G1P, OR11G2, OR11H5P, OR11H6, OR11H4, CTD-2292M16.2.1, TTC5, CTD-2292M16.7.1, CCNB1IP1, RP11-203M5.4.1, SNORA79, SNORD126, RPPH1.1, RPPH1, PARP2 RP11-203M5.2.1, TEP1, 5S_rRNA, KLHL33, RP11-203M5.6.1, OSGEP, RP11-203M5.7.1, APEX1, TMEM55B, PNP, RP11-203M5.8.1, RNASE10, RP11-14J7.3.1, RP11-14J7.2.1, RNASE9, RNASE11, AL163195.3.1, RP11-14J7.6.1, RNASE12.1, RNASE12 PKHD1* |
| 6 | 51858157 | 52238923 | *MIR206 RP11-771D21.2.1, MIR133B, IL17A, IL17F, SLC25A20P1, MCM3, PAQR8, DNAH6* |
| 2 | 84702898 | 85301342 | *DUXAP1, LDHAP7, C2orf89, AC010087.1, RPL12P18, TMSB10, AC022210.2.1, KCMF1, RP11-60H5.1.1, AC078974.2.1* |
| 13 | 100772901 | 101089435 | *PCCA, RPL15P18* |

Fluid Intelligence

| Chr | Region start (bp) | Region end (bp) | Known genes in region |
| --- | --- | --- | --- |
| 5 | 126711782 | 127335370 | *MEGF10, CTD-2195M15.1.1, HNRNPKP1, PRRC1, CTXN3, CTC-548H10.2.1, CTC-228N24.1.1, RP11-483H11.1.1, CTC-228N24.2.1, CTC-228N24.3.1,* |
| 6 | 39140691 | 39378453 | *KCNK5* |
| 6 | 39236400 | 39493104 | *KCNK17, KCNK16, KIF6, RP1-137F1.3.1* |
| 13 | 65117143 | 65633593 | *LGMNP1* |
| 6 | 740414 | 1013400 | *RP5-1077H22.2.1, RP5-1077H22.1.1* |
| 11 | 102565882 | 102978790 | *MMP27, MMP8, RP11-725K16.4.1, AP000619.5.1, AP000647.3.1, MMP10, MMP1, AP000619.6.1, MMP3, MMP12, RP11-725K16.2.1, U7* |
| 11 | 102824059 | 103220693 | *MMP13, RP11-690D19.1.1, RP11-690D19.3.1, DCUN1D5, DYNC2H1* |
| 9 | 78430995 | 78767837 | *PCSK5, AL359253.1* |
| 3 | 101162780 | 101999012 | *SENP7, Y_RNA, snoU13, FAM172BP, RG9MTD1, PCNP, RP11-234A1.1.1, RP11-454H13.1.1, U6, Y_RNA, Y_RNA, ZBTB11, Y_RNA, RP11-454H13.5.1. RPL24, RP11-454H13.6.1, CEP97, FAM55C, RP11-49I4.3.1, NFKBIZ, RP11-221J22.1.1, Y_RNA, RP11-221J22.2.1, ZPLD1* |
| 5 | 33703559 | 34034521 | *ADAMTS12, U6, RXFP3, SLC45A2, RP11-1084J3.1.1, AMACR, C1QTNF3, RP11-1084J3.3.1* |
| 2 | 151358558 | 151655394.5 | *AC104777.2.1, AC104777.3.1, AC104777.1.1, AC104777.4.1* |
| 5 | 127010643 | 127650653 | *CTC-228N24.1.1, RP11-483H11.1.1, CTC-228N24.2.1, CTC-228N24.3.1, KDELC1P1, SLC12A2, FBN2* |

Cognitive change

| Chr | Region start (bp) | Region end (bp) | Known genes in region |
| --- | --- | --- | --- |
| 4 | 53606097 | 54158143 | *ERVMER34-1, SNORA26, Y_RNA, RP11-752D24.3.1, RASL11B, SCFD2, RP11-752D24.2.1, U6* |
| 5 | 90960003 | 91404141 | *IQGAP1, CRTC3, CTD-3065B20.2.1, CTD-3065B20.3.1, RP11-387D10.2.1, CTD-3065B20.1.1, RP11-387D10.3.1, BLM, SNORD18, CTD-3094K11.1.1* |
| 6 | 740414 | 1013400 | *RP5-1077H22.2.1, RP5-1077H22.1.1* |
| 4 | 62441864 | 63300488 | *LPHN3, RP11-621K7.1.1, Y_RNA, RP11-84A1.3.1, RP11-84A1.1.1, RP11-30P21.1.1, RP11-30P21.2.1* |
| 6 | 891665 | 1138987 | *RP5-1077H22.2.1, RP5-1077H22.1.1, AL033381.1* |
| 6 | 12418779 | 12930959 | *RPL15P3, RP11-125M16.1.1, PHACTR1, AL354680.1* |
| 2 | 237734083 | 238123037 | *COPS8, AC105760.3.1, AC105760.2.1, AC107079.1.1, U6* |
| 13 | 98189341 | 98677491 | *RP11-120E13.1.1, snoU13, RPL7AP61, IPO5, FTLP8* |
| 14 | 64270578 | 64666246 | *Y_RNA, SYNE2, ESR2, MIR548H1* |
| 6 | 88043140 | 88678348 | *C6orf162, C6orf163, RP1-102H19.6.1, C6orf164, C6orf165, RP3-382I10.2.1, RP3-382I10.3.1, ST13P16, SLC35A1, U6, RARS2, ORC3, RP3-486L4.3.1, AKIRIN2, AKIRIN2-AS1, snoU13, AL138919.2, AL138919.1, Y_RNA* |
| 4 | 148617678 | 149254898 | *ARHGAP10, NR3C2, RP11-76G10.1.1, RN5S165, AC093835.1* |

Gene names obtained from Ensembl genome build 37

For pathway analysis, lists of genes within the top regions for each trait were extracted using the BioMart tool of the Ensembl database at http://www.ensembl.org/biomart/martview/. Ensembl gene ids were entered into the Database for Annotation, Visualization and Integrated Discovery (DAVID) software to test for significantly enriched pathways[[10](#_ENREF_10)] using the functional annotation tool. The software did not identify any significantly enriched KEGG pathways. For fluid intelligence DAVID software did identify the Alzheimer disease-presenilin pathway defined in the Panther database [www.pantherdb.org/genes](http://www.pantherdb.org/genes) as significantly enriched. This is due to six matrix metellopeptidase genes. We stress, however, that these are only exploratory analyses. Although the most significant regions span plausible candidate genes, some of which are described below, re-sequencing data would be required to disentangle the fine scale contribution of linked genes and pathways.

*Cognitive change*

The window on chromosome 4 spans *LPHN3* which has been linked with neurological function, in particular ADHD and response to stimulants[[11](#_ENREF_11)]. The window on chromosome 13 spans *IPO5* linked to schizophrenia[[12](#_ENREF_12)]. The region of greatest significance for cognitive change on chromosome 15 contains the *CRTC3* gene which has been associated with cognitive factors associated with processing speed[[13](#_ENREF_13)].

Three regions on chromosome 6 are in the top ten for decline and for fluid intelligence. The chromosomal analysis is suggestive that the variation attributed to chromosome 6 is caused by factors that lie outside known genes although the window spans a large region including 5 known genes. The single SNP analysis is suggestive of significance with a *P-*value of 2.69E-06 associated with rs1764142 at position 956570 bp which lies within an uncharacterised gene. This region is also one of the top regions for fluid intelligence with the same SNP showing the strongest association with the trait on chromosome 6.

*Fluid Intelligence*

For fluid intelligence the most significant region was on chromosome 5. Within the region is the *MEGF10* receptor for amyloid beta uptake[[14](#_ENREF_14)] and *CTXN3* a kidney and brain expressed protein identified in GWAS using brain imaging for schizophrenia associated brain activity linked to neurodevelopment and stress response[[15](#_ENREF_15)].

*Crystallised Intelligence*

The most significant region for crystallised intelligence is positioned on chromosome 10 and contains neuregulin 3 (*NRG3)* which has been associated with developmental delay, cognitive impairment, autism, schizophrenia and neuroblast formation[[16](#_ENREF_16),[17](#_ENREF_17)]. Also on chromosome 10 is *PTPLA* a gene associated with Alzheimer’s disease[[18](#_ENREF_18)]. On Chromosome 14 a putative candidate is *TEP1* previously associated with longevity[[19](#_ENREF_19)].

#### References

#### 1. Scottish-Council-Research-Education (1933) The intelligence of Scottish children: a national survey of an age-group. Scottish Council for Research in Education.

#### 2. Deary IJ, Whiteman MC, Starr JM, Whalley LJ, Fox HC (2004) The impact of childhood intelligence on later life: Following up the Scottish Mental Surveys of 1932 and 1947. Journal of Personality and Social Psychology 86: 130-147.

#### 3. Raven JC, Court, J.H., Raven, J. (1977) Manual for Raven's Progressive Matrices and Vocabulary Scales. London, UK: HK Lewis.

#### 4. Wechsler D (1987) Wechsler Memory Scale–Revised. San Antonio, TX Psychological Corporation.

#### 5. Lezak MD, Howieson, D.B., Loring, D.W. (2004) Neuropsychological Assessment. Oxford, UK: Oxford University Press.

#### 6. Wechsler D (1997) WAIS-III UK Administration and Scoring Manual. London, UK: Psychological Corporation.

#### 7. Deary IJ, Whalley, L.J., Starr, J.M. (2009) A Lifetime of Intelligence: Follow-up Studies of the Scottish Mental Surveys of 1932 and 1947. Washington, DC: American Psychological Association.

#### 8. Scottish-Council-Research-Education (1949) The Trend of Scottish Intelligence. Scottish Council for Research in Education.

#### 9. Wechsler D (1981) Wechsler Adult Intelligence Scale-Revised. New York: Psychological Corporation.

#### 10. Huang DW, Sherman BT, Lempicki RA (2009) Systematic and integrative analysis of large gene lists using DAVID bioinformatics resources. Nature Protocols 4: 44-57.

#### 11. Arcos-Burgos M, Jain M, Acosta MT, Shively S, Stanescu H, et al. (2010) A common variant of the latrophilin 3 gene, LPHN3, confers susceptibility to ADHD and predicts effectiveness of stimulant medication. Molecular Psychiatry 15: 1053-1066.

#### 12. Wang ZQ, Liu Y, Wu N, Xu Q, Jin SZ, et al. (2011) Genetic and functional study of the IPO5 gene in schizophrenia. Psychiatry Research 187: 460-461.

#### 13. Luciano M, Hansell NK, Lahti J, Davies G, Medland SE, et al. (2011) Whole genome association scan for genetic polymorphisms influencing information processing speed. Biological Psychology 86: 193-202.

#### 14. Singh TD, Park SY, Bae JS, Yun Y, Bae YC, et al. (2010) MEGF10 functions as a receptor for the uptake of amyloid-beta. Febs Letters 584: 3936-3942.

#### 15. Potkin SG, Turner JA, Guffanti G, Lakatos A, Fallon JH, et al. (2009) A Genome-Wide Association Study of Schizophrenia Using Brain Activation as a Quantitative Phenotype. Schizophrenia Bulletin 35: 96-108.

#### 16. Kao WT, Wang YH, Kleinman JE, Lipska BK, Hyde TM, et al. (2010) Common genetic variation in Neuregulin 3 (NRG3) influences risk for schizophrenia and impacts NRG3 expression in human brain. Proceedings of the National Academy of Sciences of the United States of America 107: 15619-15624.

#### 17. Morar B, Dragovic M, Waters FAV, Chandler D, Kalaydjieva L, et al. (2011) Neuregulin 3 (NRG3) as a susceptibility gene in a schizophrenia subtype with florid delusions and relatively spared cognition. Molecular Psychiatry 16: 860-866.

#### 18. Liang XY, Slifer M, Martin ER, Schnetz-Boutaud N, Bartlett J, et al. (2009) Genomic Convergence to Identify Candidate Genes for Alzheimer Disease on Chromosome 10. Human Mutation 30: 463-471.

#### 19. Zee RYL, Ridker PM, Chasman DI (2011) Genetic variants in eleven telomere-associated genes and the risk of incident cardio/cerebrovascular disease: The Women's Genome Health Study. Clinica Chimica Acta 412: 199-202.
